# Sleep Quality and Mood State in Resident Physicians during COVID-19 Pandemic

**DOI:** 10.3390/ijerph18158023

**Published:** 2021-07-29

**Authors:** Chiara Costa, Michele Teodoro, Giusi Briguglio, Ermanno Vitale, Federica Giambò, Giuliano Indelicato, Elvira Micali, Sebastiano Italia, Concettina Fenga

**Affiliations:** 1 Clinical and Experimental Medicine Department, University of Messina, 98125 Messina, Italy; ccosta@unime.it; 2 Department of Biomedical and Dental Sciences and Morphofunctional Imaging, Occupational Medicine Section, University of Messina, 98125 Messina, Italy; michele.teodoro@unime.it (M.T.); giusi.briguglio@unime.it (G.B.); ermannovitale@gmail.com (E.V.); federicagiambo@gmail.com (F.G.); giulianoindelicato90@gmail.com (G.I.); elvira.micali@unime.it (E.M.); cfenga@unime.it (C.F.)

**Keywords:** sleep quality, daytime sleepiness, mood disturbances, COVID-19, resident physicians

## Abstract

Since the novel coronavirus (SARS-CoV-2) has spread worldwide, healthcare workers—resident physicians in particular—have been hugely involved in facing the COVID-19 pandemic, experiencing unprecedented challenges in fighting the disease. We aimed to evaluate the prevalence of poor sleep quality, daytime sleepiness, and alterations in mood state profiles in this category. This cross-sectional study, conducted in 2020, enrolled 119 subjects from a university hospital in southern Italy. Epworth Sleepiness Scale (ESS), Pittsburgh Sleep Quality Index (PSQI), and Profile of Mood States (POMS) questionnaires were administered to physicians divided into four areas: anesthesiology, medicine, service, and surgery. In the overall sample, approximately 45% reported poor sleep quality, although only nine subjects (8%) reported an ESS score that suggested excessive daytime sleepiness. Alterations in mood profiles were also observed; the Vigor and Fatigue factors were the most altered. In particular, anesthesiologists seem to be the most affected category, showing a profound decrease in Vigor with a concomitant increase in Fatigue. Considering the possible consequences of the COVID-19 pandemic, preventive measures should be adopted, especially those aimed at facilitating a better turnover of physicians, optimizing the working schedule, and improving the organization of work.

## 1. Introduction

Since the novel coronavirus (SARS-CoV-2, first described in China) has spread worldwide, healthcare workers have been hugely involved in facing the COVID-19 pandemic [1]. Moreover, healthcare employees have experienced extraordinary challenges in fighting the disease, including close contact with patients, exposure to high viral loads, relocation of clinical tasks beyond their usual assignments, and critical scarcity of personal protective equipment (PPE) [2]. This rapidly expanding illness has revealed a severe lack of healthcare workers to face the mass hospitalization of patients across the whole world. Among the different measures that have been put into place, the most frequent has been the reorganization of hospital staff, including all medical personnel involved in the care of COVID-19 patients, regardless of their occupational background [3]. In general, resident physicians represent the most vulnerable workers due to their incomplete professional expertise, notwithstanding they constitute a considerable component of the healthcare workforce [4].

Resident physicians’ health and wellbeing are well documented as crucial factors in the current healthcare workforce’s efficiency and performance. Health status is influenced by various positive and negative pressures, such as work commitment, job gratification, work-related stress, and organizational support [5]. In particular, during the COVID-19 pandemic, trainees are more susceptible to suffering from mood drops because of the sudden work overload in healthcare settings, which are frequently stressful and distinguished by more strenuous shifts, fluctuating work hours, and a high-pressure atmosphere [6]. Moreover, precisely owing to these working conditions, healthcare workers are more likely to report a high prevalence of sleep disturbances with consequential psychological disorders affecting daily activities [7]. Therefore, it is possible to observe a reduction in professional performance among medical staff with severe consequences, such as mistakes due to fatigue leading to adverse clinical outcomes [8,9].

With consequent isolation and social distancing, the pandemic has changed interactions among the general population and affected residents’ approaches to patients, working schedules, and the organization of traineeships. The majority of the studies on trainees have focused on the impact of COVID-19 on residency programs [10,11,12,13], but only a few have investigated their health status and psychological wellbeing [14,15].

Our previous research, conducted before the COVID-19 outbreak, reported absence of sleep disturbances and minor mood disorders in a similar group of residents [16]. Predictably, we have hypothesized that changes in workload, tasks, organization, and schedule consequent to the pandemic may have played a role in altering these parameters.

Under these premises, we aim to evaluate sleep quality, daytime sleepiness, and alterations in mood state profiles in resident physicians during the COVID-19 pandemic, along with sociodemographic, health, lifestyle, and work-related factors.

## 2. Materials and Methods

### 2.1. Study Design and Population

This cross-sectional study was conducted from February to August 2020 among the departments of the university hospital “Policlinico Vittorio Emanuele” in Catania (Italy).

Participants were resident physicians who worked during the COVID-19 pandemic, either employed in night shifts or not. In the Italian working organization, shift work involves 38 h per week, and night shift work is described as a period of at least three uninterrupted hours between 0 a.m. and 5 a.m. According to current Italian legislation, residents are included in a compulsory medical surveillance program; on that occasion, they were invited to participate in the investigation activity without any reward. All the residents who accepted voluntary participation in the survey provided written informed consent. This study was carried out in accordance with the Declaration of Helsinki’s ethical standards. Being part of the mandatory occupational health surveillance, the study needed no formal approval by the local ethics committee. A total of 183 subjects were invited to participate in the current investigation.

Moreover, we divided the physicians into different groups by stratifying them into four areas: anesthesiology, medicine, service, and surgery. Anesthesiology residents manage general and loco-regional anesthesia, intensive care units, and palliative care. Physicians in the area of medicine mainly focus on clinical activities and instrumental investigations such as ultrasound scans, digestive endoscopy, and respiratory function exams. During the training course, a period in the emergency department is also required. The area of surgery includes several disciplines such as general surgery, neurosurgery, and orthopedics. The activities mainly involve operating room interventions, including laparoscopic and arthroscopic procedures and pre- and post-operative hospitalization management. The area of service includes the residents of occupational medicine, forensic medicine, hygiene, and preventive medicine. Their activities comprise compulsory health surveillance, research facilities activity and also environmental and biological monitoring.

### 2.2. Measures

Data collected involved the sociodemographic characteristics of gender, age, marital status, and parenthood. Health and lifestyle factors included body mass index (BMI), smoking habit, alcohol intake, and chronic drug use. Work-related factors consisted of year in the residency program, night shifts, and working hours per week.

We administered three standardized questionnaires during a regular morning shift: Epworth Sleepiness Scale (ESS), Pittsburgh Sleep Quality Index (PSQI), and Profile of Mood States (POMS).

The Epworth Sleepiness Scale (ESS) comprises eight questions (each scored from 0 to 3, with a total score ranging from 0 to 24). This questionnaire assesses the overall level of daytime sleepiness, considered abnormal when the score is >10 [17].

The Pittsburgh Sleep Quality Index (PSQI) comprises 7 components (each scored from 0 to 3, with a total score ranging from 0 to 21) and provides an individual measure of sleep quality. While a score >5 is considered to suggest the presence of sleep disorders, a score >10 is indicative of very poor sleep quality [18].

The Italian version of the Profile of Mood States (POMS) is made up of 58 items that define six binary mood factors: Tension-Anxiety (T), Depression-Dejection (D), Anger-Hostility (A), Vigor-Activity (V), Fatigue-Inertia (F), Confusion-Bewilderment (C). Each question is ranked on a 5-point Likert scale from 0 (not at all) to 4 (extremely). Each factor includes a variable number of items; all item scores, composing the same factor, are summed to calculate each factor score. The total POMS score is calculated by subtracting the Vigor factor from the sum of Anxiety, Depression, Anger, Fatigue and Confusion. Finally, the raw scores are transformed into T standard scores based on normal distribution data deriving from the reference population [19].

### 2.3. Statistical Analysis

Statistical analysis was performed using Graph Pad Prism 8 (GraphPad Software, San Diego, CA, USA). Descriptive analyses were performed for all variables. Categorical variables were expressed as frequency and proportion, whilst continuous variables were expressed as mean and standard deviation. To determine differences between groups in categorical variables, we used chi-square tests and Fisher’s exact tests, as appropriate. The differences in continuous variables were evaluated using the one-way analysis of variance (ANOVA) and the Kruskal–Wallis test, followed by post hoc comparison utilizing Dunn’s test. Computing Pearson *r* coefficients tested the possible association between all variables. Moreover, in order to individuate possible predictive variables, we applied a binary logistic regression for PSQI and ESS, while we used a generalized linear model for the POMS factors, both on the overall study population and each specialty macro-area. *p* values < 0.05 were considered statistically significant.

## 3. Results

A total of 119 subjects out of the 183 invited accepted to participate in the study and completed the survey (an acceptance rate of 65%). A detailed sample description is summarized in Table 1.

The whole sample was stratified into four areas: anesthesiology, medicine, surgery, and service (29, 33, 32, and 25 subjects, respectively). The study population consisted of 63 women (52.9%) and 56 men (47.1%), aged 27–47 years, with a mean of 30.7 ± 3 years. We found statistically significant differences among the four groups in gender and age; in fact, while 80% of surgeons were men, this proportion was inverted in the area of anesthesiology. In addition, the area of surgery showed a greater mean age (32.27 ± 6.2 years). Among the 119 physicians, 104 (87.4%) were unmarried and only 15 (12.6%) were married, while 12 (10.1%) participants had children. Concerning health status and lifestyle factors, 20 (19.8%) subjects took drugs chronically and the mean BMI was 23.6 ± 3.3. Moreover, 36.1% (43) of the individuals were smokers, and 47.1% (56) reported moderate alcohol consumption. Regarding careers and work-related factors, 48 (40.3%) subjects were attending the first or second year of their medical specialization course. In the whole sample, 63% (75 individuals) of the physicians worked night shifts; in particular, all the anesthesiologists worked at night, in contrast to the area of service in which only 28% of residents performed night shifts. The mean duration of working time was 41.4 ± 6.9 h per week, without any statistically significant differences among the groups.

A detailed description of the mean and standard deviation of the ESS, PSQI, and POMS questionnaire scores is reported in Table 2, for both the overall study sample and specific areas.

The total sample ESS score was 6.47 ± 2.98, but anesthesiologists and surgeons reported higher values (7.17 ± 2.98 and 7.12 ± 3.05, respectively) than the other areas. Moreover, PSQI scores showed only a slight difference between the four groups, presenting in the whole study population a score of 6.20 ± 2.83. Additionally, we proceeded to stratify the four groups according to ESS and PSQI cut-offs, as shown in Table 3.

The overall prevalence of daytime sleepiness was 7.56%, while sleep disturbances were reported by 35.45% of all participants and 10% of subjects suffered from very poor sleep quality. Although the anesthesiologists reported the highest prevalence of daytime sleepiness (approximately 14%) and the surgeons reported the highest occurrence of poor sleep quality (56%), no statistically significant differences were observed between the groups.

**Figure 1:**
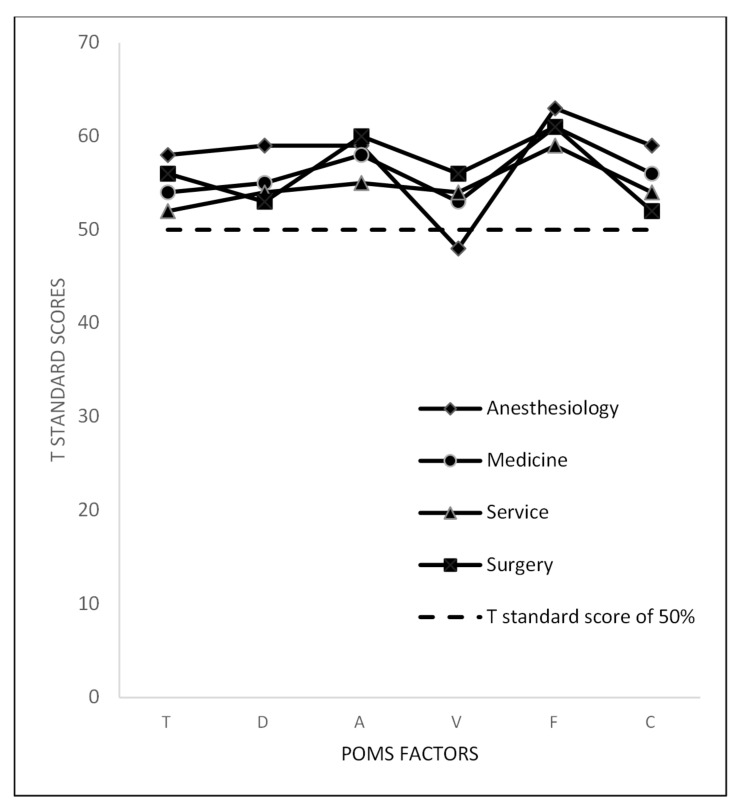
Mean POMS scores in residents, stratified according to specialty macro-area.

Considering the mood state subscale scores, although the anesthesiologists showed higher values in the POMS negative factors (14.14 ± 5.95 in Tension, 15.62 ± 13.18 in Depression, 14.21 ± 11.03 in Anger, 11.76 ± 4.30 in Fatigue, 11.79 ± 4.69 in Confusion), we did not observe any statistically significant differences between the groups (see Table 2), except for the Vigor (V) subscale, in which the surgeons reported the highest value and the anesthesiologists the lowest ones. Furthermore, to better understand the POMS scores, we have reported the distribution of T standard scores of the six subscales among the four groups in a graph, as displayed in Figure 1.

We also investigated all the possible associations between the questionnaire outcomes and the sociodemographic, health status, lifestyle, and work-related variables through a Spearman correlation analysis. In the overall sample, sleep quality did not show any statistically significant association. Although the PSQI was negatively associated with age (*r* = −0.357, *p*-value = 0.045) in the area of service, it was positively associated with having children (*r* = 0.366, *p*-value = 0.036) and with chronic use of drugs (*r* = 0.507, *p*-value = 0.010) in the areas of medicine and surgery, respectively. Moreover, PSQI scores were negatively associated with work hours per week (*r* = −0.389, *p*-value = 0.037) in the area of anesthesiology. Concerning daily sleepiness, ESS scores were positively associated with night shifts (*r* = 0.205, *p*-value = 0.025) in the overall sample and with smoking habit (*r* = 0.374, *p*-value = 0.032) in the area of medicine. Considering the interrelation between sleep quality and daily sleepiness, the PSQI and ESS scores showed a positive association (*r* = 0.237, *p*-value = 0.009). However, dividing into different groups, this association is only statistically significant among residents in the area of surgery (*r* = 0.432, *p*-value = 0.031).

Moreover, the binary logistic regression for PSQI and ESS did not reveal any predictive variables. On the contrary, the generalized linear model for the POMS factors only showed different predictors when distinctly considering the four specialization areas. Although analysis of the medicine group did not display any statistically significant results, in the anesthesiology group, we found the year of residency program to be a predictive variable of Depression (B −7.10; 95% CI −13.25–−0.95; *p*-value 0.024), Anger (B −5.33; 95% CI −10.63–−0.02; *p*-value 0.049), and Fatigue (B −2.51; 95% CI −4.16–−0.89; *p*-value 0.003). We found similar findings for the service group for the year of residency program: Tension (B −2.18; 95% CI −3.46–−0.89; *p*-value 0.001), Depression (B −7.10; 95% CI −13.25–−0.95; *p*-value 0.024), Anger (B −2.90; 95% CI −5.36–−0.43; *p*-value 0.021), Fatigue (B −1.63; 95% CI −2.71–−0.56; *p*-value 0.003); and for the surgery group: Tension (B −3.88; 95% CI −6.11–−1.66; *p*-value 0.001), Depression (B −5.00; 95% CI −8.21–−1.80; *p*-value 0.002), Vigor (B 3.55; 95% CI 1.59–5.50; *p*-value < 0.001), Fatigue (B −2.96; 95% CI −4.86–−1.06; *p*-value 0.002). Furthermore, night shifts acted as another important predictive variable for the service group: Tension (B −4.26; 95% CI −7.46–−1.07; *p*-value 0.009), Depression (B −9.18; 95% CI −15.18–−3.17; *p*-value 0.003), Anger (B −9.88; 95% CI −15.99–−3.77; *p*-value 0.002), Fatigue (B −2.84; 95% CI −5.51–−0.17; *p*-value 0.037); and for the surgery group: Tension (B 8.50; 95% CI 2.82–14.17; *p*-value 0.003), Depression (B 13.33; 95% CI 5.16–21.50; *p*-value 0.001), Vigor (B −9.64; 95% CI −14.62–−4.66; *p*-value < 0.001), Fatigue (B 6.42; 95% CI 1.59–11.25; *p*-value 0.009).

## 4. Discussion

In the current study, we evaluated sleep quality, daytime sleepiness, and alterations in mood state profiles in resident physicians during the COVID-19 pandemic.

Our results showed a high prevalence of important alterations in sleep quality and mood states in the resident physicians who participated in this investigation. Even though we found poor sleep quality in almost half of the subjects in our sample, only a few subjects showed excessive daily sleepiness. Concerning mood state profiles, the results revealed a dip in Vigor and a peak in Fatigue, especially in anesthesiology trainees.

Conversely to the results of our previous investigation performed on a comparable population of resident physicians, in which no sleep disturbances were detected [16], in the current research, over a third of subjects reported poor sleep quality and 10% very poor sleep quality. These results parallel another study conducted among Saudi physicians, in which the prevalence of sleep disorders was approximately 44% during the COVID-19 pandemic [20]. Moreover, unexpectedly, our results highlighted that daily workers showed worse sleep quality than night shift staff. However, no statistically significant differences were found, contrariwise to the existing literature regarding healthcare employees [21] and other working categories in the pre-COVID era [22]. We can hypothesize that daily workers were subjected to increased workloads similarly to night shift residents. Therefore, these profound changes in work organization and environment may have contributed to circadian rhythm dysregulation, thus worsening sleep quality. Surprisingly, better sleep quality was associated with long working hours per week among anesthesiologists, contrary to another study in which the number of workdays was negatively related to sleep quality [23]. Anesthetists represented the category most involved in managing COVID-19 patients; therefore, they were burdened by increased workloads and reorganization in work shifts. Nonetheless, those with better sleep quality displayed good work performance, working for a more extended period, albeit showing increased fatigue levels.

Additionally, despite the data regarding sleep quality, only 8% reported an ESS score > 10 (which would suggest excessive daytime sleepiness), showing a positive association with poor sleep quality, in agreement with another study conducted on healthcare workers in which only 6% reported high ESS scores [24]. These data may be justified by the excessive load of new tasks assigned, leading to a continuous increase in the attention threshold and constant stimulation of the sleep/wake cycle, resulting in a low prevalence of daytime sleepiness.

Emotional involvement, assessed by the POMS questionnaire, highlighted borderline values above average in all residents belonging to the different specialty areas. In particular, the Vigor (V) and Fatigue (F) factors were the most affected, especially in the area of anesthesiology. Indeed, it is noteworthy that anesthetists exhibited the highest values for the F factor, presenting a V factor below the average. This dip could suggest inactivity or inertia, but if combined with the high levels of fatigue, it represents a symptom of an excessive workload. During the pandemic, anesthesiologists and intensive care unit staff were the most overwhelmed by the challenging treatments required by COVID-19 patients, according to a recent meta-analysis [25]. Moreover, the analysis of other factors composing the sphere of affection outlined a resident profile characterized by irritability (A factor), disorientation (C factor), depression (D factor) and worry (T factor), in agreement with the findings of other researches that demonstrated an increase in anxiety and depression levels in healthcare workers [26], especially in intensive care unit or sub-intensive COVID-19 unit staff [27]. We can explain these findings through the individual conditions due to social isolation and the changes in the work environment, which may have acted as precursors in raising stress levels with consequent repercussions on the factors mentioned above, negatively influencing mood profiles. In fact, the continuous state of emergency that has caused an ever-increasing number of hospitalizations may have contributed to increasing the state of anxiety, especially in its performance component. Moreover, this young population missed the equilibrium between work and spare time to improve their social life, due to lockdown policies, thus suffering social marginalization.

Notably, in the early stages of the pandemic, there was a generalized state of bewilderment in the management of COVID-19 patients due to the paucity of guidelines. We could hypothesize that this situation may have increased the frustration which resulted in a rise in anger levels, showing the highest scores in surgeons, but a different explanation for this aspect can be found for these residents. In fact, the first phase of the pandemic led to a drastic reduction in surgical activities, which may have increased frustration and therefore anger, with a consequent stoppage in the learning curves [28].

The generalized linear model results reveal that being enrolled in the first year of the residency program predicts negative alterations in mood states. We can hypothesize that residents who started their medical training during the pandemic were most affected by the impact of the new course beginning, which was partially modified by the epidemiological trend. Moreover, night shifts were related to unfavorable mood disorders in the area of surgery, paralleling the results of other studies conducted before and during the pandemic [29,30]. Thus, we cannot assume that the COVID-19 outbreak acted as the cause of mood disorders, but it may have played a pejorative role. Unexpectedly, in the area of service, we found an opposite trend; indeed, not working during the nights is a predictive variable of a worse mood profile. In accordance with Italian training programs, residents’ schedules in the area of service does not include night shifts, but these physicians have been recruited to support COVID wards due to the lack of medical personnel during the pandemic. We suggest that these profound changes in work organization may have played a significant role in altering emotional involvement. In particular, participating in the management of the emergency and contributing to the improvement of the epidemic situation could have played a role in improving emotional well-being, whilst not being involved could have triggered feelings of frustration, with adverse repercussions on mood factors.

Globally, the mood states observed in the study population can be explained by the long duration of the emergency, with greater alterations on both perceived Fatigue and Vigor, indicating profiles with a high risk of decreased work performance and residents’ psychophysical well-being.

To our knowledge, only a few studies have assessed sleep quality, daytime sleepiness, and mood disturbances among resident physicians during the COVID-19 pandemic. Our investigation could provide new insights into the burden of these alterations in young physicians. The current study also has some limitations. Firstly, we might have overestimated the prevalence of sleep disorders and alterations in mood profiles because subjects who voluntarily participate in the survey might be more conscious of their mental health status than those who chose not to enroll. Secondly, although we administrated validated questionnaires, assessing sleep disorders and daytime sleepiness through subjective means may not mirror the participants’ authentic characteristics. Thirdly, in a previously cited research [16], we investigated another group of residents with parallel sociodemographic characteristics and work tasks assigned by nationally harmonized guidelines, despite being enrolled in a different hospital; under these premises, we have hypothesized that the well-known changes in workload, work tasks, turn-over, and working time since the beginning of the pandemic might have played a role in altering the considered outcomes compared to pre-pandemic study population; nonetheless, we are aware that this study design does not allow to establish a cause–effect relation.

Furthermore, non-linear statistical models could have been applied to explore further associations between the considered outcomes and the sociodemographic and work-related variables.

## 5. Conclusions

Although further studies are needed, we can hypothesize that the COVID-19 pandemic has exacerbated existing problems negatively influencing resident physicians’ health status by increasing sleep disturbances and altering mood states. In particular, anesthesiologists showed the most significant alterations, resulting in a profound decrease in vigor and a concomitant increase in fatigue. Occupational health physicians may play a strategic role during the pandemic; they could identify critical alterations of mood early and properly manage these, minimizing the risk for future burnout and maximizing the benefits for the workers.

Particularly in the COVID-19 pandemic situation, we suggest that specific measures should be adopted, especially those aimed at optimizing the working schedule, improving the work organization and facilitating a better turnover of physicians, especially those at the beginning of their residency program.

## Figures and Tables

**Table 1:** Sociodemographic factors and health, lifestyle, and work-related characteristics of the sample, stratified according to specialty macro-area.

	Overall	Anesthesiology	Medicine	Service	Surgery	*p*-Value
	N (%)	N (%)	N (%)	N (%)	N (%)	
Sociodemographic factors						
Total	119 (100.00)	29 (24.17)	33 (27.50)	32 (26.67)	25 (20.83)	
Gender						
Male	56 (47.06)	6 (20.69)	14 (42.42)	16 (50.00)	20 (80.00)	<0.001
Female	63 (52.94)	23 (79.31)	19 (57.58)	16 (50.00)	5 (20.00)	
Age						
Mean ± SD	30.66 ± 3.04	31.79 ± 3.59	29.45 ± 1.86	31.06 ± 3.66	32.24 ± 6.22	0.015
Range	27–47	28–47	27–34	27–41	27–35	
Marital status						
Married	15 (12.61)	3 (10.34)	3 (9.09)	7 (21.88)	2 (8.00)	0.390
Unmarried	104 (87.39)	26 (89.66)	30 (90.91)	25 (78.13)	23 (92.00)	
Parenthood						
Yes	12 (10.08)	1 (3.45)	3 (9.09)	7 (21.88)	1 (4.00)	0.099
No	107 (89.92)	28 (96.55)	30 (90.91)	25 (78.13)	24 (96.00)	
Health and lifestyle factors						
BMI						
Mean ± SD	23.55 ± 3.25	23.21 ± 3.77	23.12 ± 3.11	23.18 ± 3.00	24.98 ± 2.82	0.059
Smoking habit						
Yes	43 (36.13)	11 (37.93)	7 (21.21)	13 (40.63)	12 (48.00)	0.170
No	76 (63.87)	18 (62.07)	26 (78.79)	19 (59.38)	13 (52.00)	
Alcohol intake						
Yes	56 (47.06)	14 (48.28)	16 (48.48)	13 (40.63)	13 (52.00)	0.844
No	63 (52.94)	15 (51.72)	17 (51.52)	19 (59.38)	12 (48.00)	
Drugs						
Yes	20 (16.81)	5 (17.24)	8 (24.24)	3 (9.38)	4 (16.00)	0.475
No	99 (83.19)	24 (82.76)	25 (75.76)	29 (90.63)	21 (84.00)	
Work-related factors						
Year in residency program						
First	24 (20.00)	0 (0.00)	3 (9.09)	14 (43.75)	7 (28.00)	<0.001
Second	24 (20.00)	3 (10.34)	14 (42.42)	3 (9.38)	4 (16.00)	
Third	39 (32.50)	15 (51.72)	10 (30.30)	10 (31.25)	3 (12.00)	
Fourth	26 (21.67)	8 (27.59)	6 (18.18)	5 (15.63)	7 (28.00)	
Fifth	7 (5.83)	3 (10.34)	0 (0.00)	0 (0.00)	4 (16.00)	
Night shifts						
Yes	75 (63.03)	29 (100.00)	18 (54.55)	9 (28.13)	19 (76.00)	<0.001
No	44 (36.97)	0 (0.00)	15 (45.45)	23 (71.88)	6 (24.00)	
Hours per week						
Mean ± SD	41.39 ± 6.86	40.62 ± 4.46	42.42 ± 8.43	39.22 ± 4.13	43.68 ± 8.78	0.067

**Table 2:** Total score of ESS, PSQI, and POMS questionnaires (Mean ± SD).

	Overall	Anesthesiology	Medicine	Service	Surgery	*p*-Value
	Mean ± SD	Mean ± SD	Mean ± SD	Mean ± SD	Mean ± SD	
ESS	6.47 ± 2.98	7.17 ± 2.98	5.94 ± 2.67	5.88 ± 3.14	7.12 ± 3.05	0.233
PSQI	6.20 ± 2.83	6.03 ± 2.57	6.24 ± 3.61	6.28 ± 2.51	6.24 ± 2.47	0.790
POMS	43.09 ± 34.92	53.52 ± 40.55	43.15 ± 33.74	36.47 ± 32.36	39.40 ± 31.69	0.552
POMS factors						
Tension (T)	12.31 ± 5.65	14.14 ± 5.95	11.79 ± 5.50	10.94 ± 5.36	12.64 ± 5.57	0.197
Depression (D)	12.98 ± 10.43	15.62 ± 13.18	12.82 ± 9.51	12.09 ± 10.24	11.28 ± 7.96	0.731
Anger (A)	13.17 ± 9.53	14.21 ± 11.03	12.97 ± 8.94	11.31 ± 9.43	14.60 ± 8.68	0.560
Vigor (V)	16.71 ± 5.26	14.00 ± 4.88	16.79 ± 5.05	17.44 ± 5.27	18.84 ± 4.92	0.010
Fatigue (F)	10.95 ± 4.66	11.76 ± 4.30	11.30 ± 4.99	9.91 ± 5.05	10.88 ± 4.10	0.512
Confusion (C)	10.39 ± 4.92	11.79 ± 4.69	11.06 ± 5.19	9.66 ± 4.75	8.84 ± 4.67	0.139

Epworth Sleepiness Scale (ESS), Pittsburgh Sleep Quality Index (PSQI), Profile of Mood States (POMS).

**Table 3:** Stratification of the results of ESS and PSQI questionnaires with respect to cut-off.

	Overall	Anesthesiology	Medicine	Service	Surgery	*p*-Value
	N (%)	N (%)	N (%)	N (%)	N (%)	
ESS						
≤10	110 (92.44)	25 (86.21)	33 (100.00)	30 (93.75)	22 (88.00)	0.107
>10	9 (7.56)	4 (13.79)	0 (0.00)	2 (6.25)	3 (12.00)	
PSQI						
≤5	66 (55.46)	19 (65.52)	20 (60.61)	16 (50.00)	11 (44.00)	0.531
6–10	41 (34.45)	7 (24.14)	9 (27.27)	14 (43.75)	11 (44.00)	
>10	12 (10.08)	3 (10.34)	4 (12.12)	2 (6.25)	3 (12.00)	

Epworth Sleepiness Scale (ESS), Pittsburgh Sleep Quality Index (PSQI).

## Data Availability

The datasets generated and analyzed during the current study are available from the corresponding author on reasonable request.
